# A Case of Leishmaniasis Infantum Kala-Azar in an Immunocompetent 49-Year-Old Man

**DOI:** 10.7759/cureus.25442

**Published:** 2022-05-29

**Authors:** Carla Williams, Jessica Bass, Anshika Singh, Kelsey Diemer

**Affiliations:** 1 Department of Internal Medicine, NCH Healthcare System, Naples, USA

**Keywords:** hepatosplenomegaly, mucocutaneous, leishmaniasis, pancytopenia, miltefosine, visceral, cutaneous, fever, hepatomegaly, splenomegaly

## Abstract

An immunocompetent 45-year-old Cuban-American man presented with worsening knee pain and swelling despite antibiotic therapy. On physical examination, the patient was ill-appearing, cachectic, with a protuberant abdomen and massive splenomegaly. In addition, he had a 10 cm area of peripheral hyperemia with central necrosis in the medial left knee that was non-tender and non-fluctuant. Initial lab work demonstrated pancytopenia, hyponatremia, hypoalbuminemia, and anemia of chronic inflammation. Peripheral smear showed microcytic, hypochromic red blood cells with mild anisopoikilocytosis. and leukopenia with slight left shift and metamyelocytes. Bone marrow biopsy demonstrated amastigotes and kinetoplasts within white blood cells and extracellular space consistent with leishmaniasis. Centers for Disease Control and Prevention (CDC) testing with PCR returned positive for *Leishmaniasis infantum*. The patient received two courses of amphotericin B lipid complex (ABLC) with a 28-day course of miltefosine, which resulted in clinical improvement. This case illustrates the unique pathology that can affect immigrants and highlights the need to increase health provider awareness of foreign pathologies in areas with large migrant populations.

## Introduction

Leishmaniasis is a zoonotic disease spread through the bites of female phlebotomine sandflies. Leishmania protozoa from the Trypanosome genus live in the midgut and pharynx of sandflies thriving in rural and natural environments [1]. Cutaneous leishmaniasis is the most common form of this disease. However, the visceral type, also known as “black fever” or “kala-azar,” is the most severe form and is fatal if untreated [2]. *Leishmania (L.) infantum* and *Leishmania donovani* are usually responsible for causing visceral leishmaniasis. *L. infantum*, endemic to the Mediterranean and much of Latin America, where it is referred to as *Leishmaniasis chagasi*, is the cause of infantile visceral leishmaniasis and a cause of cutaneous leishmaniasis [3]. The infection is usually self-limited in immunocompetent hosts. The classical findings of more severe disease include fever, cachexia, hepatosplenomegaly, pancytopenia, and hypergammaglobulinemia, and symptoms of visceral Leishmaniasis occur over months to years depending on the region of infection [2].

Classified by the World Health Organization (WHO) as a neglected tropical disease and rarely reported in the United States, Leishmaniasis presents major diagnostic and therapeutic challenges. These factors make leishmaniasis a diagnostic challenge. Furthermore, treatment in the United States often represents an additional hurdle after diagnosis, as little clinical data exists for optimal treatment, and therapies carry significant toxicity [4]. This report describes a case of visceral leishmaniasis in a migrant laborer who presented with knee pain, cachexia, hepatosplenomegaly, and pancytopenia as an uncommon cause for pancytopenia within the United States.

## Case presentation

A 45-year-old Cuban-American man with no known past medical history, no alcohol consumption, and remote history of travel to Spain (Cordova region) for work returned to the emergency department with complaints of worsening medial left knee swelling and pain after completing one week of outpatient clindamycin therapy. Ten days before his initial evaluation, the patient hit his left knee on a metal bucket while at work but did not develop open wounds or abrasions. The patient used ibuprofen and acetaminophen for the pain without relief. He also endorsed progressive “flu-like symptoms," fatigue, and weight loss over the previous three years on review of systems but had not been medically evaluated regarding these medical issues.

On examination, the patient was chronically ill-appearing, cachectic with temporal wasting, peripheral wasting, and a protuberant abdomen. His blood pressure was 122/70 mmHg, pulse 98 beats per minute, temperature 36.9^o^C, and respiratory rate 18 breaths per minute. He had significant medial left knee edema with an approximately 10 cm rim of peripheral hyperemia that was non-tender to palpation and non-fluctuant with a central eschar 1 cm in diameter. He had decreased active range of motion of the left knee to 30 degrees flexion with no effusion palpated. His abdomen was firm, distended, and non-tender with massive splenomegaly.

Laboratory results were significant for pancytopenia, hypoalbuminemia, and elevated inflammatory markers (Table 1). 

**Table 1:** Laboratory results on presentation

SIGNIFICANT LABS	ON PRESENTATION	REFERENCE RANGE
Leukocytes	0.5 th/uL	4.2-10.8 th/uL
Erythrocytes	3.35 mil/uL	4-5.4 mil/uL
Hemoglobin	8.5 gm/dL	14-18 gm/dL
Platelets	78 th/uL	130-450 th/uL
Hematocrit	28.90%	42-52%
Lymphocytes	48%	24-44%
Monocytes	17%	0-15%
Eosinophils	2%	0-5%
Basophils	0.30%	0-3%
Absolute Neutrophil Count	0.26 th/uL	1.8-7%
Potassium	3.8 mmol/L	3.5-5.1 mmol/L
Sodium	131 mmol/L	136-145 mmol/L
Chloride	102 mmol/L	98-107 mmol/L
Bicarbonate	28 mmol/L	21-32 mmol/L
Blood Urea Nitrogen	12 mg/dL	7-18 mg/dL
Creatinine	0.8 mg/dL	0.6-1.3 mg/dL
Erythrocyte Sedimentation Rate	78 mm/hr	0-10 mm/hr
Lactate Dehydrogenase	138 IU/L	87-241 IU/L
C-Reactive Protein	7.9 mg/dL	< 0.03 mg/dL
Iron	12 mcg/dL	65-175 mcg/dL
Total Iron-Binding Capacity	200 mcg/dL	286-515 mcg/dL
Ferritin	174 ng/mL	26-388 ng/mL
Transferrin	140 mg/dL	200-360 mg/dL
Vitamin B12	1395 pg/mL	193-986 pg/mL
Folate	6.10 ng/mL	3.1-17.5 ng/mL
Copper	81 mcg/dL	70-175 mcg/dL
Zinc	36 mcg/dL	60-130 mcg/dL
Antinuclear Antibodies	negative	-
HIV Ab/Ag, 4th gen	non-reactive	-
Histoplasma mycelia	negative	-
Urine Histoplasma Antigen	negative	-

**Figure 1:**
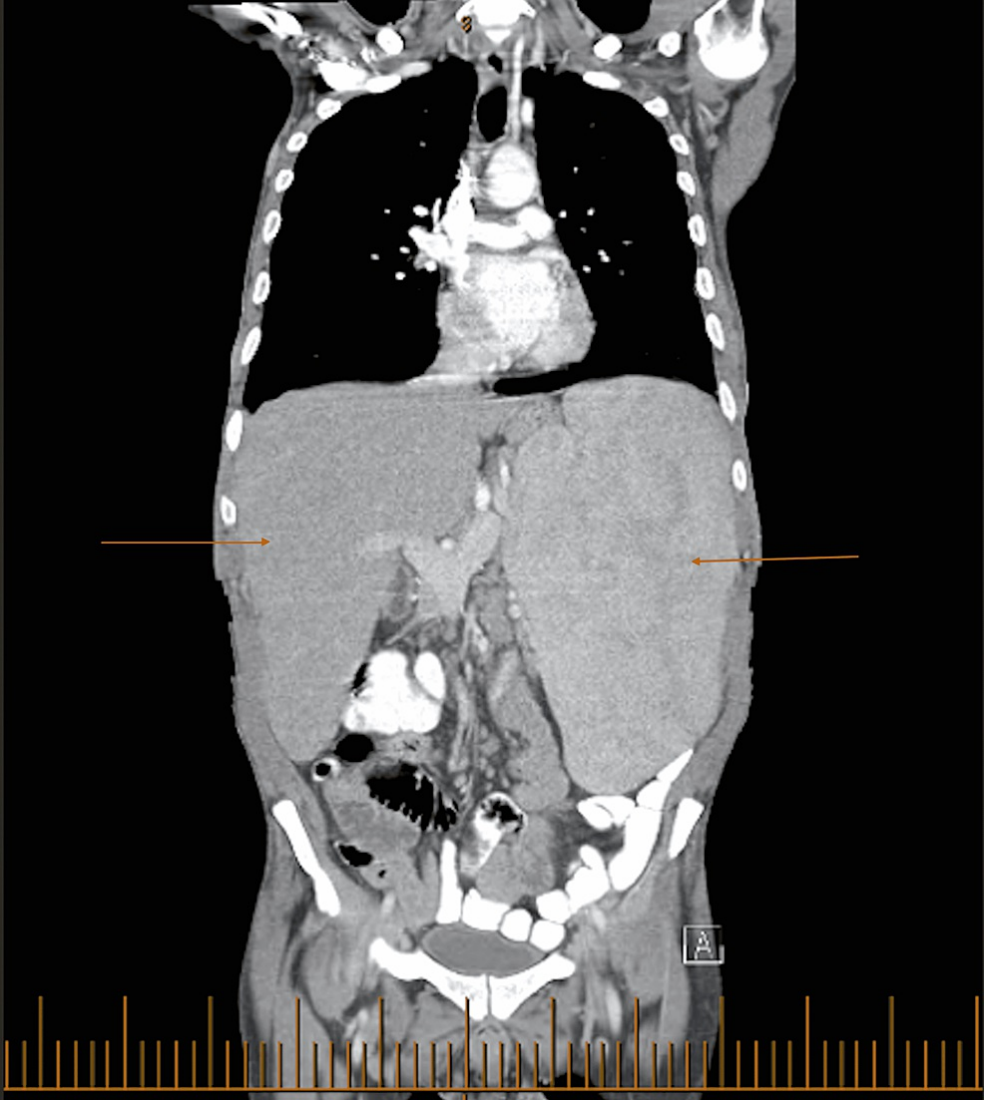
CT abdomen and pelvis without contrast revealing hepatosplenomegaly

The antinuclear antibodies screen was negative. Immunoglobulin G (IgG) was markedly elevated at 5.3 g/dL; however, serum protein electrophoresis did not reveal an M spike. A peripheral smear showed microcytic, hypochromic red blood cells with mild anisopoikilocytosis and leukocytosis with a left shift. Metamyelocytes were present, and there were no giant platelets or platelet aggregates. A CT abdomen and pelvis without IV contrast revealed hepatomegaly, splenomegaly with heterogeneous density, and a left renal hypodensity (Figure 1).

**Figure 2:**
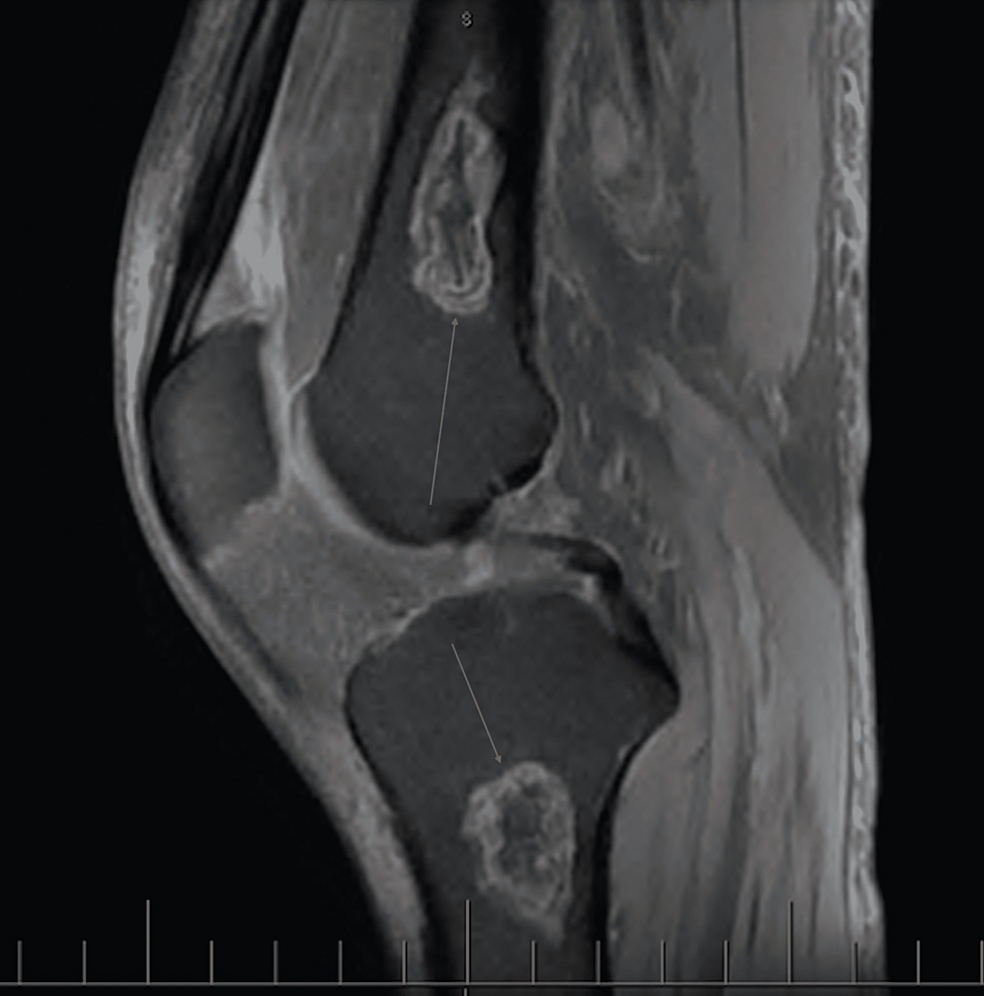
MRI of the left knee with and without contrast revealing bony involvement

MRI of the left knee with and without IV contrast revealed lesions of the bone involving the distal femur and proximal tibia and a small joint effusion (Figure 2).

A superficial knee-wound culture was positive for methicillin-resistant-staph aureus (MRSA). However, multiple blood cultures revealed no growth. Initial differential diagnoses included leukemia, lymphoma, nutritional deficiency, HIV/AIDS, tuberculosis, and histoplasmosis.

**Figure 3:**
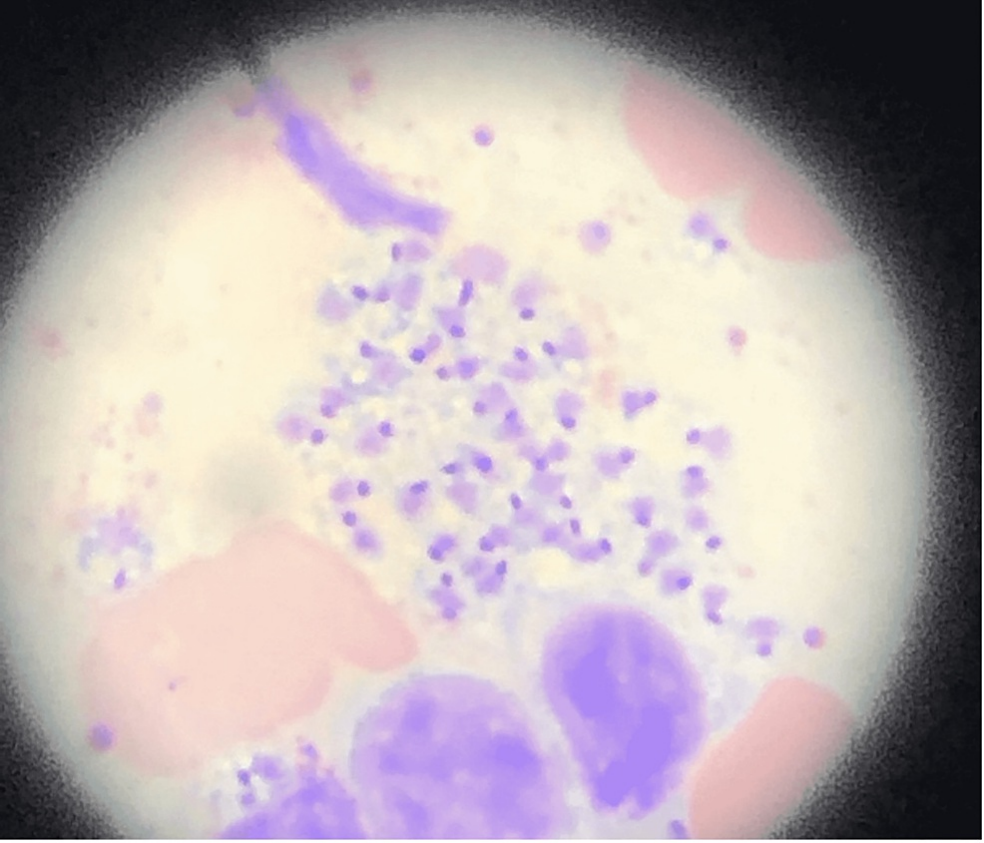
Histopathology with Giemsa staining, 100x magnification, showing small organisms with morphology suggestive of Trypanosomatidae, specifically Leishmania species both inside white cells and in extracellular space Peripheral blood with pancytopenia

The bone marrow biopsy pathology obtained to evaluate the patient’s pancytopenia revealed amastigotes and kinetoplasts inside white cells and in the extracellular space with morphological features consistent with Trypanosomatidae species, specifically leishmaniasis (Figure 3).

The Leishmania antibody was 3.12 (reference range <1.00). The diagnosis was confirmed by CDC testing with polymerase chain reaction (PCR), which was positive for *Leishmaniasis infantum*.

The patient received treatment for two weeks with amphotericin B lipid complex (ABLC), a total dose of 42 mg/kg. His symptoms initially improved, then deteriorated over approximately three months. He showed continued bone marrow infiltration of leishmaniasis five months later and was started on a 28-day course of miltefosine and a repeat ABLC regimen. He then continued with monthly infusions of ABLC at 4 mg/kg for suppression for 12 months in total. After nine months of continued therapy, splenomegaly was no longer detectable on physical examination; the patient gained weight and clinically improved. In addition, his leukocyte count, hemoglobin, and platelets normalized as did his immunoglobulin levels.

## Discussion

The incidence of visceral leishmaniasis (VL) in the United States is low but increasing [5]. While VL is endemic in over 60 countries, the highest disease burden is shared by six countries, including Bangladesh, Brazil, Ethiopia, India, South Sudan, and Sudan [6]. Our patient was born in Cuba and immigrated to the United States. VL is not endemic in either of these countries, and no autochthonous cases have been reported in the USA and Cuba [7]. He reported intermittent travel to southern Spain (Cordova region) for work. In Spain, domesticated dogs have been recognized as a reservoir for L. infantum. Per WHO, leishmaniasis is hypo-endemic in Spain, with less than 0.41 cases every 100,000 inhabitants. Between 2009 and 2012, an outbreak of 446 cases was recorded in Madrid, where *L. infantum* was identified as the causative agent [8]. Our patient was in Spain much later in 2016-2018. But given the same species isolated from his bone marrow, we suspect that is where he potentially acquired the infection. Amongst the Mediterranean countries, Spain is known to have a higher prevalence of Leishmania-HIV coinfection, with a large proportion of the high-risk population being IV drug users (IVDU) [8]. The lack of contact with dogs, HIV-negative status, and no history of IV drug use further makes this case an epidemiological mystery.

Leishmaniasis’ clinical presentation is broadly classified as cutaneous, mucocutaneous, and visceral [2]. Despite exhibiting clinical findings consistent with typical VL, such as cachexia, pancytopenia, hepatosplenomegaly, and hypergammaglobulinemia, this diagnosis was not one of the initial differentials likely because healthcare providers in non-endemic countries have little experience in recognizing Leishmaniasis. Pancytopenia and hepatosplenomegaly in an otherwise healthy adult are more commonly linked with leukemia, lymphoma, chronic myelodysplastic syndromes, HIV, tuberculosis, and histoplasmosis. In this case, HIV was negative upon multiple checks, Histoplasma testing was negative, and bone marrow biopsy did not show evidence of myelodysplastic disease or neoplastic process but rather amastigotes and kinetoplasts consistent with Trypanosomatidae species, resulting in this diagnosis. This serves as a reminder for clinicians in non-endemic countries, particularly in tropical areas or places with large migrant populations, to not overlook rare parasitic infections as a cause of pancytopenia and hepatosplenomegaly. VL has been reported amongst US soldiers deployed to Saudi Arabia and Iran since Operation Desert Shield from 1990 through 2016 [9]. A study found 19.5% of 200 deployers to have asymptomatic VL [10]. This rise in travel-related VL cases in the US further necessitates the need for clinicians to familiarize themselves with its diagnosis and management. The diagnosis of *L. infantum *at early stages can be confirmed with PCR testing. The molecular diagnosis is the best for the cutaneous leishmaniasis as the culturing of the organism is difficult and the immunological assay fails to detect antibodies created against the parasite. 

The cutaneous lesion noted on this patient’s knee represents an interesting point for discussion as cutaneous manifestations are much more common than visceral disease. The lesions appear as erythematous papules, which eventually develop into a round ulcerated lesion with a thick nodular border with sharp edges [2]. While cutaneous lesions can often be the first sign of disease, post-Kala-Azar dermal leishmaniasis (PKDL) is a well-reported phenomenon in long-standing illnesses. It is often characterized by nodular densities in multiple skin lesions [11]. The knee lesion described in our patient matches what can be seen in cutaneous leishmaniasis. However, it must be noted that the time period for PKDL varies by region and therefore must be taken into account when considering leishmaniasis in the differential diagnosis. Though the synovial fluid culture grew MRSA, the clinical significance is unclear as the culture was superficial and could merely represent colonization. Additionally, blood cultures were negative, making colonization that much more likely. The protozoa can be seen under the microscope in samples collected by scraping the borders of the cutaneous lesions in both ulcerated and non-ulcerated forms [12]. Skin scrapings from this patient’s knee to examine for the parasite were not performed, precluding a definitive diagnosis of a cutaneous leishmaniasis lesion, though clinical suspicion is high.

The patient’s clinical course was complicated though it ultimately ended favorably. He exhibited multiple clinical features that have been associated with increased mortality risk such as jaundice, anemia, malnutrition, long duration of illness, and splenomegaly [2]. Treatment with ABLC, as is recommended first-line by the CDC, was first performed. However, the patient relapsed over several months with a repeat bone marrow biopsy showing no improvement from the initial specimen. Little data are available regarding the treatment of VL caused by *L. infantum*. Miltefosine is best studied in *L. donovani* but is considered off-label for *L. infantum *[4]. This patient responded well to miltefosine with chronic ABLC suppression.

## Conclusions

With less than 100,000 new cases of visceral leishmaniasis annually and the vast majority of those being in endemic areas, early recognition of this parasitic infection, particularly in places like the United States, is challenging. However, recent data indicate that the incidence of leishmaniasis in the United States is increasing. The goal of this case report is to increase awareness of leishmaniasis as a potential cause of pancytopenia and hepatosplenomegaly, particularly in areas with large immigrant populations. This case is unique, as it illustrates a case of visceral leishmaniasis in an otherwise immunocompetent person. It also highlights the importance of thorough history-taking and the need for more rapid testing of uncommon microbial organisms when evaluating immigrant populations, even those not considered immunocompromised.
